# Fatal *Cronobacter sakazakii* Sequence Type 494 Meningitis in a Newborn, Brazil

**DOI:** 10.3201/eid2410.180373

**Published:** 2018-10

**Authors:** Cláudia Elizabeth Volpe Chaves, Marcelo Luiz Lima Brandão, Mara Luci Gonçalves Galiz Lacerda, Caroline Aparecida Barbosa Coelho Rocha, Sandra Maria do Valle Leone de Oliveira, Tânia Cristina Parpinelli, Luiza Vasconcellos, Stephen James Forsythe, Anamaria Mello Miranda Paniago

**Affiliations:** National Institute of Quality Control in Health of Oswaldo Cruz Foundation, Rio de Janeiro, Brazil (C.E.V. Chaves, M.L.L. Brandão, L. Vasconcellos);; Federal University of Mato Grosso do Sul, Mato Grosso do Sul, Brazil (C.E.V. Chaves, S.M. do Valle Leone de Oliveira, A.M.M. Paniago);; Regional Hospital of Mato Grosso do Sul, Mato Grosso do Sul (M.L.G. Galiz Lacerda, C.A.B. Coelho Rocha, T.C. Parpinelli);; foodmicrobe.com, Adams Hill, Nottingham, UK (S.J. Forsythe)

**Keywords:** infection, neonate, *Cronobacter*, epidemiology, bacteria, meningitis/encephalitis, Brazil, *Cronobacter sakazakii*

## Abstract

We describe a case of infection with *Cronobacter sakazakii* sequence type 494 causing bacteremia and meningitis in a hospitalized late premature infant in Brazil. We conducted microbiological analyses on samples of powdered infant formula from the same batch as formula ingested by the infant but could not identify the source of contamination.

In September 2017, a healthy boy was born at 35 weeks’ gestation in Brazil. The newborn was fed breast milk and reconstituted powdered infant formula (PIF) while in the hospital. On postnatal day 4, he began sleeping more than usual and experienced hypoactivity, pallor, jaundice, seizures, metabolic acidosis, and finally respiratory insufficiency, necessitating mechanical ventilation and empiric treatment with cefepime and ampicillin. We obtained 2 blood cultures on postnatal day 4 that yielded *Cronobacter* spp. with resistance to cephalothin and cefoxitin, intermediate resistance to nitrofurantoin, and susceptibility to other antimicrobial drugs, including cefepime and ampicillin. A transfontanel ultrasound on postnatal day 6 showed grade 2 periintraventricular hemorrhage with hypoxic-ischemic lesions. Subsequent computed tomography and nuclear magnetic resonance (NMR) imaging revealed biparietal cerebral abscess (Figure). Culture of the cerebral abscess on postnatal day 33 yielded *Cronobacter* spp. that had the same pattern of antimicrobial drug susceptibility as that found in blood isolates. Because of the patient’s progressive clinical deterioration, we changed the antimicrobial therapy to meropenem on postnatal day 10; however, the infant failed to improve, and he died on postnatal day 46.

**Figure:**
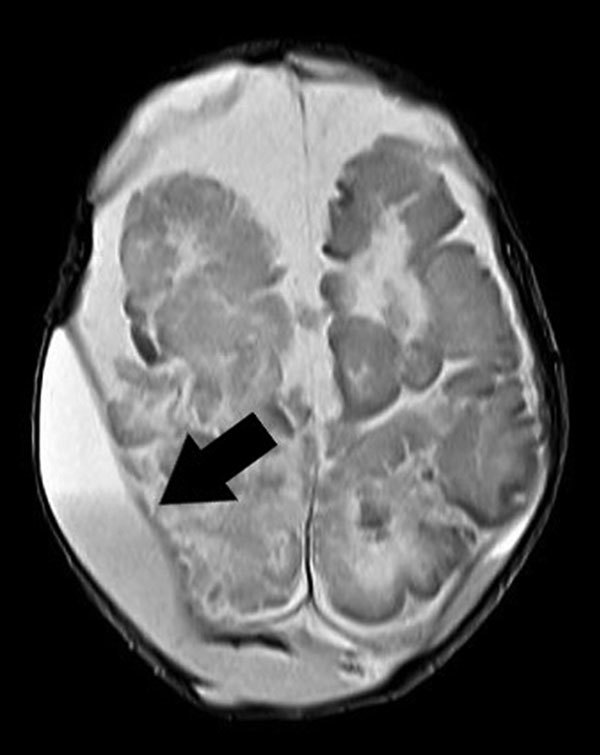
Brain nuclear magnetic resonance image of a newborn with *Cronobacter sakazakii* sequence type 494 meningitis, Brazil. Extradural collections are visible in both parietal regions. Arrow indicates the more pronounced extradural collection, measuring ≈1.8 cm, in the right parietal region.

The mother’s pregnancy was uncomplicated except for a urinary tract infection (UTI), for which she received cephalexin, in the third trimester. She experienced another UTI caused by *Enterococcus faecalis* and endometritis shortly after giving birth and underwent endometrial curettage and received ampicillin/sulbactam. We did not identify *Cronobacter* spp. in the urine or endometrial curettage material.

We were unable to analyze a sample from PIF container, the contents of which had been used. Because contaminated PIF from opened cans has been identified as the vehicle in nearly all infant *Cronobacter* infections in the past decade for which a source has been found (*1*), this lack of testing is probably the most significant limitation of this investigation. Subsequently, we sent an unopened can of the PIF from the same lot consumed by the newborn to Nacional Institute of Quality Control in Health from Oswaldo Cruz Foundation (INCQS/Fiocruz), a public laboratory in the Brazilian System of Sanitary Surveillance, where the PIF was analyzed in accordance with 2 standard procedures (*2**,**3*). Neither method recovered *Cronobacter.*

According to the hospital’s standard procedure, all PIF was reconstituted with potable water that was heated >70°C, cooled, and used immediately. However, we could not trace the total time between the reconstitution of the PIF, the time it was maintained during cooling, and the subsequent feeding to the newborn, because the hospital does not have procedures for recording this process. 

Ten newborns, 6 of whom were preterm infants, had been fed from the same lot of PIF. We followed the infants clinically during their hospital stay, and none showed signs or symptoms of *Cronobacter* infection. 

We did not obtain swab specimens for surveillance. We collected environmental samples from the newborn’s location after birth and from formula preparation equipment for microbiological tests in the hospital approximately 3 weeks after illness onset. However, several cleaning and disinfection procedures had been performed, limiting our chances to detect the pathogen in these samples. We collected 1 rectal swab from the newborn brother of the patient on postnatal day 4. We streaked samples onto the surface of ChromID CPS agar (bioMérieux, Rio de Janeiro, Brazil), and test results were negative for *Cronobacter*. However, this method is not specific for *Cronobacter* isolation.

Contaminated PIF and expressed breast milk have been epidemiologically linked with *Cronobacter* infections in neonates (*1**,**4*), and cases in Brazil have been reported in the literature (*5**,**6*). The magnitude of *Cronobacter* disease in Brazil is unclear, partly because it is not a compulsory notifiable disease. The most recent reported cases occurred in 2013, when *C. malonaticus* sequence types (ST) 394 and ST440 were responsible for bacteremia in 3 neonates, and the source of contamination was not identified (*5**,**7*). 

In our study, the analyzed PIF did not show *Cronobacter* contamination. In addition, the method of PIF preparation used in the hospital (using water >70°C) would probably inactivate any *Cronobacter* present in the PIF. Because we analyzed only 1 sample, it is possible that we did not detect *Cronobacter* because contamination was not homogeneous across the lot or was below the limit of detection for our methods. We recommend the use of sterile liquid infant formulas in the hospital for patients in neonatal intensive care units unless there is no suitable alternative.

We used the *Cronobacter* MLST Database (http://pubmlst.org/cronobacter) to perform multilocus sequence typing on the 3 *Cronobacter* isolates we detected (*8*). We identified the strains as *C. sakazakii* ST494, an ST which is not in any of the recognized *C. sakazakii* clonal complex (CC) pathovars, such as *C. sakazakii* CC4, which is strongly associated with neonatal meningitis (*9*). 

*Cronobacter* bacteria can cause severe meningitis, resulting in brain abscess formation. Virulence studies of *C. sakazakii* ST494 strains are needed to elucidate their pathogenicity and to compare with *C. sakazakii* CC4 strains.
